# Exploring the anti-inflammatory effects of *Lavandula stoechas* L. extract on ovalbumin-induced acute asthma in BALB/c mice

**DOI:** 10.22038/AJP.2024.25175

**Published:** 2025

**Authors:** Nafiseh Erfanian, Faezeh Fazlpour, Hossein Safarpour, Sayyedeh Fatemeh Askari, Mohsen Foadoddini, Saeed Nasseri

**Affiliations:** 1 * Cellular and Molecular Research Center, Birjand University of Medical Sciences, Birjand, Iran*; 2 * Student Research Committee, School of Medicine, Birjand University of Medical Sciences, Birjand, Iran*; 3 *Pharmaceutical Science Research Center, Birjand University of Medical Sciences, Birjand, Iran*; 4 *Department of Pharmacognosy and Traditional Pharmacy, School of Pharmacy, Birjand University of Medical Sciences, Birjand, Iran*; 5 * Cardiovascular Diseases Research Center, Birjand University of Medical Sciences, Birjand, Iran*

**Keywords:** Acute asthma, Anti-inflammatory, Lavandula stoechas L, Ovalbumin

## Abstract

**Objective:**

Asthma is an inflammatory disease of the respiratory system affecting over 300 million people worldwide. *Lavandula stoechas *L. (*L. stoechas*) has traditionally been used to manage inflammatory diseases and against multiple medical conditions. In this study, we aimed to explore the anti-inflammatory effects of the hydro-alcoholic extract of *L. stoechas* in a mice model of acute asthma.

**Materials and Methods:**

Thirty-five male BALB/c mice were placed into five distinct study groups: (1) control, (2) ovalbumin (OVA) + Al(OH)3, (3) OVA + *L. stoechas* (200 mg/kg), (4) OVA + *L. stoechas* (300 mg/kg) and (5) OVA + dexamethasone. Sensitization of the mice involved intraperitoneal administration of 75 µg OVA + 2mg Al(OH)3 on days 1 and 8. Subsequently, between days 15 and 17, the mice underwent intranasal challenges with 50 µg of OVA. On days 13–18, the mice were administered either *L. stoechas* (200 and 300 mg/kg) orally or dexamethasone intraperitoneally (used as a positive control). On day 19, both bronchoalveolar lavage (BAL) fluid and lung tissue samples were collected for biochemical and immuno-histological analyses.

**Results:**

The *L. stoechas* extract-treated groups displayed notable reductions in histological alterations and inflammatory cell infiltration, surpassing the effects observed in the OVA group. Moreover, the *L. stoechas* treatment group exhibited lowered *TNF-α* and *IL-6* expression levels.

**Conclusion:**

Our results demonstrated the potential of *L. stoechas *as an anti-inflammatory agent in acute asthma.

## Introduction

Asthma, a prevalent global clinical condition affecting approximately 300 million individuals, presents significant challenges in terms of patient risk and healthcare costs associated with acute episodes (Majellano et al., 2023; Shikama et al., 2023; Mehta et al., 2019). Traditionally, asthma management decisions hinge upon clinical assessments and respiratory function evaluations (Xie et al., 2023; Jesenak et al., 2017). A notable characteristic of asthma involves elevated eosinophil counts in both peripheral blood and airway secretions, correlating with disease severity and heightened bronchial responsiveness (Muniz‐Junqueira et al., 2013; Sørensen et al., 2023). Eosinophils contribute to inflammation through the release of inflammatory substances, leading to mucus production and airway constriction (Sockrider and Fussner, 2020; Gautier and Charpin, 2017). Guideline-recommended treatment for acute asthma includes medications like oxygen, beta2-agonists, anticholinergics, and corticosteroids (Al Qahtani, 2023). Systemic corticosteroids, administered intramuscularly, intravenously, or orally, have been effective in alleviating symptoms and reversing asthma's pathological processes (Maspero et al., 2023). However, long-term use of corticosteroids is associated with adverse effects such as infections and persistent conditions (Maspero et al., 2023; Park and Cho, 2023). Seeking alternatives, attention has turned to natural remedies like *Lavandula stoechas *L. (*L. stoechas*) renowned for its diverse medicinal attributes encompassing analgesic, anti-inflammatory, antidepressant, sedative, muscle relaxant, anticonvulsant, antibacterial, and antispasmodic effects (Miraj, 2016). *L. stoechas*, a member of the Lamiaceae family, is known for its medicinal properties (Shamabadi et al., 2023; Miraj, 2016). Its aerial parts contain compounds with potent anti-inflammatory effects, making it a promising therapy for asthma and other allergic conditions (Sirohi and Jain, 2019; Ez zoubi et al., 2020). Given its anti-inflammatory and antispasmodic properties, *L. stoechas* holds promise as a potential treatment for asthma. While some exploration has been undertaken on other *Lavandula* species like *Lavandula angustifolia* (*L. angustifolia*) and *Lavandula dentata* (*L. dentata*) regarding their influence on asthma and inflammatory conditions, research into the efficacy of *L. stoechas* extract for asthma in animal models remains limited (Sirohi et al., 2019; Yassine et al., 2016; Shamsi et al., 2019). The present study delves into the anti-inflammatory potential of *L. stoechas* extract in the context of an acute asthma model using BALB/c mice. Our objective is to illuminate the potential of *L. stoechas* as a natural remedy for asthma, thereby contributing to the expansion of alternative treatment options for this prevalent condition.

## Materials and Methods

### Preparation of alcoholic extract

The extraction process involved employing both digestion and maceration techniques. To begin, the aerial parts of the plant were finely ground. Subsequently, 50 g of the powdered plant material was blended with a water-ethanol solution in a 7:3 ratio, resulting in a total volume of 1500 ml. The mixture underwent agitation at a temperature of 40℃ for 72 hr. Following this, the resultant extract was filtered through Whatman filter paper and then concentrated using a rotary evaporator. Finally, for the purposes of preservation and safeguarding against heat and moisture, the concentrated extract underwent freeze-drying and was subsequently stored within a sealed container at a temperature of 4℃ (Aboutaleb et al., 2019; Rahmah et al., 2019).

### Animals

A total of thirty-five male BALB/c mice, (25-30 g, 8 to 10 weeks old), were included. These mice were maintained under consistent conditions, residing within an environment characterized by a 12-hr light-dark cycle and a temperature ranging between 20 to 24 C°. This research adhered to ethical principles and guidelines. The Birjand University of Medical Science ethics committee in Iran provided approval with the designated ethics ID IR.BUMS.REC.1401.075.

### OVA-induced mouse model of acute asthma

**Figure 1:**
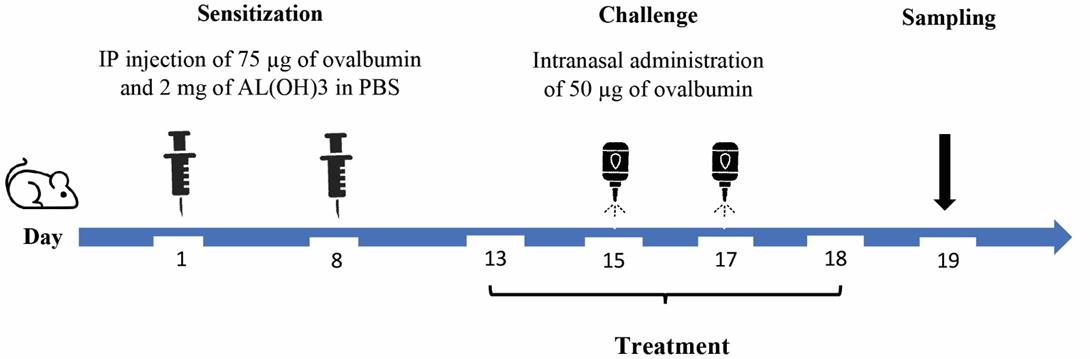
Schematic diagram of experimental design

Male BALB/c mice were methodically assigned to five distinct study groups as follows: control, ovalbumin (OVA) + Al(OH)_3_, OVA + *L. stoechas* extracts (at doses of 200 and 300 mg/kg), and OVA + dexamethasone. The sensitization process involved the administration of 75 µg of OVA and 2 mg of Al(OH)_3_ in phosphate-buffered saline (PBS) via intraperitoneal injection on days 1 and 8. Subsequently, during the period spanning days 15 to 17, the mice received intranasal challenges involving the administration of 50 µg of OVA. Between days 13 and 18, the mice were carefully assigned to one of three treatment regimens: either *L. stoechas* extract at doses of 200 mg/kg or 300 mg/kg (administered orally, twice daily) (Almohawes Z and Alruhaimi H 2019; Husseini Y et al. 2016), or dexamethasone (4 mg/kg via intraperitoneal injection, twice daily) (Gurusamy et al., 2016) (Figure 1).

### Samples collecting and cell counting

On the 19th day, mice underwent anesthesia using a combination of ketamine (80 mg/kg) and xylazine (10 mg/kg) (Shaban et al., 2023) (Figure 1). To collect bronchoalveolar lavage (BAL) fluid, a 20-gauge angiocatheter was employed. This involved instilling 0.5 ml of sterile PBS into the mouse lung, a process that was repeated three times. The collected BAL fluid was then subjected to centrifugation at 1200 rpm for 10 min at 4°C. The resulting supernatant was carefully preserved at -80°C. Total cell counts were determined using a hemocytometer. To perform differential cell counts, BAL fluid cell smears were stained with the Hema-color for Microscopy Staining Kit (IVD, Merck, Germany), and the counts were established based on morphological criteria.

### Lung histology

The upper right lobe of lung tissues was meticulously fixed in a solution of 10% formalin, followed by embedding in paraffin and sectioned into slices measuring 5 μm thick. After the deparaffinization process, slides were stained using hematoxylin and eosin. Employing light microscopy, a thorough assessment of morphological alterations in the lungs was conducted, and the extent of cellular inflammatory infiltration was quantified. The grading system for lung injury was based on specific criteria including neutrophil infiltration, edema, disorganization of lung parenchyma, and hemorrhage. This system assigned a numeric score to each criterion, reflecting the severity of lung damage. The scores were as follows: 

0 = normal, 1 = mild, 2 = moderate, 3 = severe, and 4 = very severe (Barut et al., 2016). 

Notably, higher scores corresponded to more pronounced lung injury. 

### Reverse‑transcription PCR

Quantitative assessment of mRNA expression levels for *TNF-α* and *IL-6* within lung tissues was conducted using the quantitative reverse transcription polymerase chain reaction (qRT-PCR) technique. Briefly, total RNA was extracted from the lung tissues using a commercially available kit (Parstous, Tehran, Iran). Subsequent cDNA synthesis was carried out using a high-capacity cDNA Reverse Transcription kit (Parstous, Tehran, Iran), utilizing 2 mg of RNA as the starting material. In each qRT-PCR reaction, 50 ng of cDNA was combined with the Cyber Green gene expression master mix kit (Amplicon, Denmark). The designed primers specifically targeted *TNF-α *and mice *IL-6* (Table 1). To ensure robust normalization, all data were standardized against the endogenous control eukaryotic 18S rRNA for each individual sample. PCR amplifications were meticulously executed in triplicate using the ABI Step One™ Real-Time PCR System (Applied Biosystems, Foster City, CA). 

### Statistical analysis

In the statistical analysis section, we utilized ANOVA (Analysis of Variance) to analyze the quantitative data. For pairwise comparisons, we employed Tukey's post hoc test. Additionally, to analyze qualitative data, we used the chi-square test. The results are presented as the mean±standard error of the mean (SEM). Moreover, Minitab Statistical Software was employed to assess differences with a significance level set at p<0.05.

## Results

### L. stoechas extract reduced infiltration of inflammatory cells in BAL fluid

**Figure 2:**
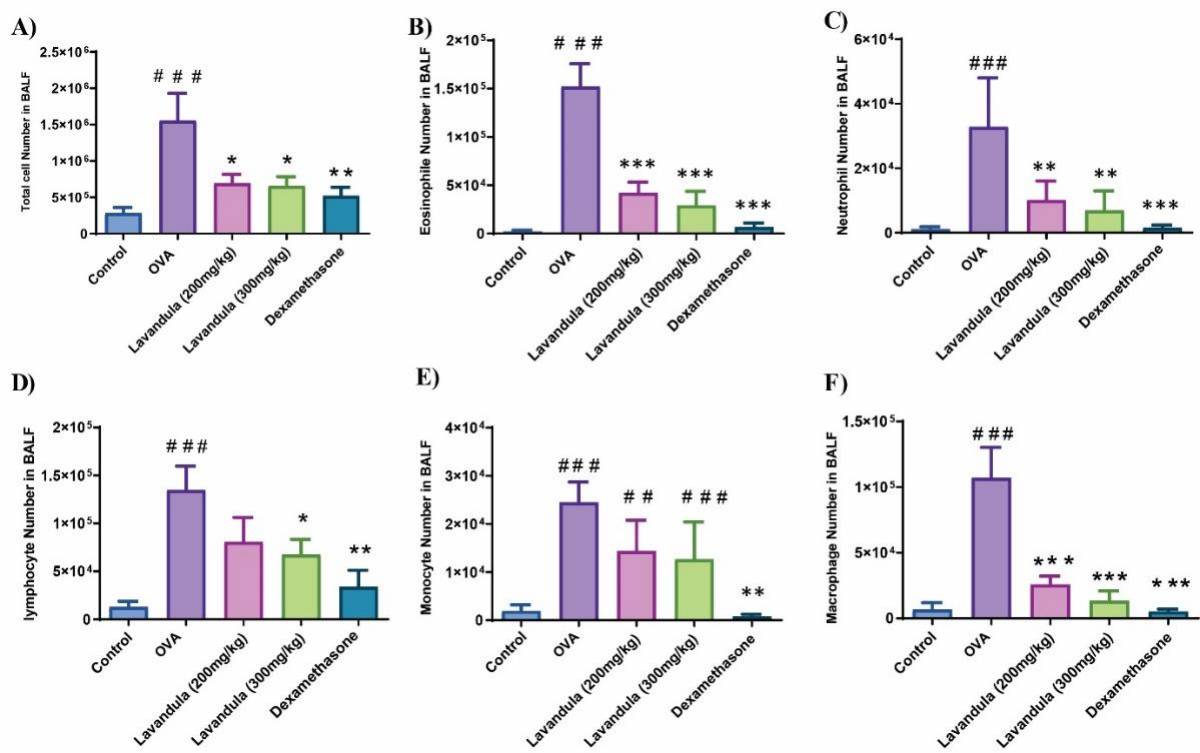
Effects of Lavandula stoechas L. (L. stoechas) (at doses of 200 and 300 mg/kg, i.p) on bronchoalveolar lavage (BAL) fluid total and differential cell counts in OVA-induced acute asthma mice. Notes: Mice were sensitized with OVA with injections on days 1 and 8, followed by intranasal injections on days 15-17. Then, 24 hr later, mice were euthanized, and BAL fluid was collected for further evaluation. Control and OVA groups did not receive any treatment. The other three groups received L. stoechas (200 and 300 mg/kg, i.p.) or Dexamethasone (4 mg/kg, i.p.). (A-F) Total inflammatory cells, eosinophils, neutrophils, lymphocytes, monocytes, and macrophages. The number of mice/groups: 7, #p-value vs. control, *p-value vs. OVA, (#p<0.05, ##p<0.01, ###p<0.001 and *p<0.05, **p<0.01, ***p<0.001). The values are shown as means ± standard error of the mean. BAL: bronchoalveolar lavage and OVA: ovalbumin.

The BAL fluid findings illustrated in Figure 2 demonstrate an increased presence of various cell types in the OVA group, including total cell count, eosinophils, neutrophils, lymphocytes, monocytes, and macrophages, when compared to the control animals. Conversely, the groups treated with *L. stoechas* at both dosage levels displayed a significant decrease in the counts of the aforementioned cell types compared to the OVA group. Additionally, the dexamethasone-treated group exhibited a noteworthy reduction in the count of all cell types compared to the OVA group.

### L. stoechas extract reduced histopathological changes in the lungs

**Figure 3:**
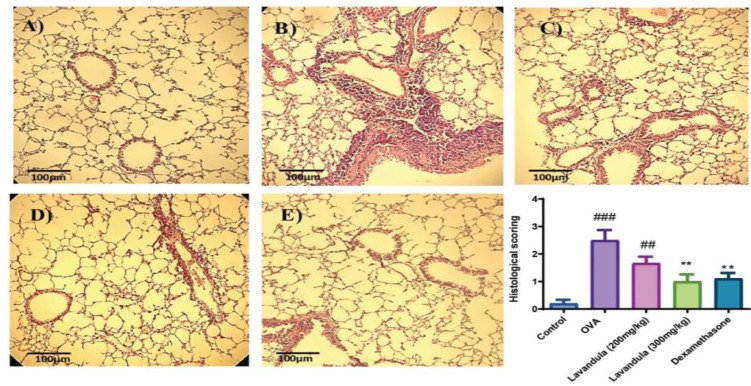
Histopathological changes in the (A) control, (B) OVA, (C) Lavandula stoechas L. (L. stoechas) doses of 200 mg/kg, (D) L. stoechas doses of 300 mg/kg, and (E) Dexamethasone-treated groups. Notes: Mice were sensitized with OVA with injections on days 1 and 8, followed by intranasal injections on days 15 to 17. Then, 24 hr later, mice were euthanized, and BAL fluid was collected for further evaluations. The number of mice per group: 7, #p-value vs. control, *p-value vs. OVA, (##p<0.01, ###p<0.001 and **p<0.01). The values are shown as means ± standard error of the mean. BAL: bronchoalveolar lavage and OVA: ovalbumin.

Histological examinations exposed pathological structural alterations induced by OVA, alongside the pronounced accumulation of polymorphonuclear leukocytes in lung tissue. As depicted in Figure 3, pulmonary specimens from the control group exhibited a regular structure without any evidence of inflammatory cell infiltration or hemorrhaging when observed under a light microscope (Figure 3a). In contrast, the OVA group demonstrated signs of airway congestion, thickening of alveolar walls, infiltration of inflammatory cells within the interstitial spaces, edema, and hemorrhage. Notably, both dexamethasone and *L. stoechas* extracts (at doses of 200 and 300 mg/kg) exhibited substantial protective effects in comparison to OVA-induced mice.

### L. stoechas extract reduced the expression levels of IL-6 and TNF-α

**Figure 4:**
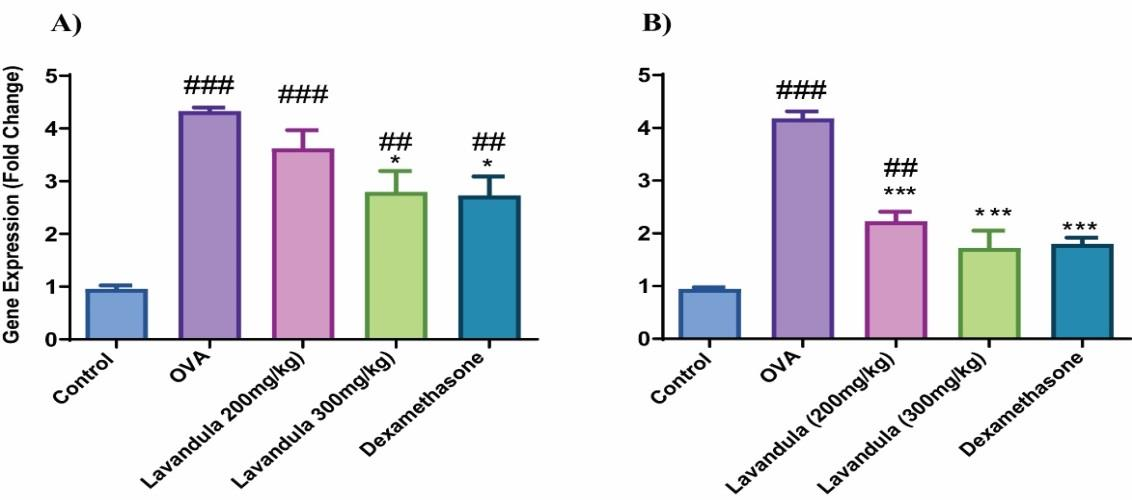
Gene expression levels IL-6 (A) and TNF-α (B) in the control, OVA, Lavandula stoechas L. (L. stoechas) (200 and 300 mg/kg doses), and dexamethasone-treated groups. The Y-axis presents the fold change. Values are reported as mean and standard deviation. The relative expression levels of IL-6 and TNF-α were calculated using the 2-ΔΔCT method with β-actin as an endogenous control. The number of mice per group: 7, #p-value vs. control, *p-value vs. OVA, (##p<0.01, ###p<0.001 and *p<0.05, ***p<0.001).

We assessed the expression levels of *IL-6* and *TNF-α* using RT-qPCR. Our findings revealed a significant upregulation of *IL-6* (p<0.001) and *TNF-α* genes (p<0.001) in the OVA samples as opposed to the control group. In contrast, the groups treated with *L. stoechas* displayed a noteworthy decrease in the expression of *IL-6* (p<0.05) and *TNF-α* genes (p<0.001) when compared to the OVA group (Figure 4).

## Discussion

In this study, our objective was to assess the inhibitory effects of *L. stoechas* on airway inflammation using a murine model of acute lung inflammation. In our initial study, we have shown that *L. stoechas* is able to reduce inflammatory cells in mice sensitized to OVA (Fazlpour et al., 2024). The present research was undertaken to further expand our understanding regarding the inhibitory effects of *L. stoechas* on OVA-sensitized animals. 

Mice subjected to OVA exposure showed a significant increase in the expression of *IL-6* and *TNF-α* genes in tissue samples, accompanied by recruitment of inflammatory cells, as indicated by analysis of BAL fluid samples. Remarkably, administration of *L. stoechas* effectively attenuated the degree of cellular infiltration into the airways, as evidenced by a notable reduction in total cell counts within the BAL fluid samples. Additionally, *L. stoechas* demonstrated efficacy in mitigating airway remodeling. 

Asthma, characterized by reduced expiratory flow and objective measures of lung function, arises from concentric smooth muscle contraction, airway wall inflammation, and luminal blockage due to mucus accumulation. Importantly, the core pathophysiologic mechanisms of asthma exacerbations might diverge based on distinct patient profiles (Wu et al., 2015; Jesenak et al., 2023). The airway epithelium, constituting the initial point of contact for inhaled agents, plays a pivotal role. Epithelial cells discharge various agents that nonspecifically safeguard the respiratory tract from microbial assaults. Furthermore, these cells generate a spectrum of mediators to mobilize inflammatory cells to sites of inflammation (Loxham et al., 2014; Ju et al., 2011). *L. stoechas*, a member of the Lamiaceae family, is a widespread plant species across diverse geographical regions. This plant is renowned for its diverse medicinal attributes and holds considerable significance (Shamabadi et al., 2023; Miraj, 2016). The aerial components of *L. stoechas* are enriched with essential compounds of alkaloids, flavonoids, glycosides, steroids, diterpenes, saponins, and tannins, collectively contributing to potent anti-inflammatory characteristics. As such, *L. stoechas* emerges as a promising therapeutic for managing allergic complications such as asthma (Sirohi et al., 2019; Ez Zoubi et al., 2020). 

The anti-inflammatory properties of the Lamiaceae family have been well-documented. *L. dentata* extract, another closely related family member of Lamiaceae, exhibited a noteworthy reduction in total white blood cell (WBC) count, indicating its potential anti-inflammatory effects (Almohawes and Alruhaimi, 2019). Additionally, *L. angustifolia* essential oil exhibited beneficial effects in mitigating allergic airway inflammation and mucus cell hyperplasia in a mouse model of asthma (Ueno-Iio et al., 2014). *L. angustifolia'*s anti-edematogenic and anti-inflammatory properties have been investigated as well (Barut et al., 2016), and its extract has been shown to reduce asthma inflammation (Khodadoost et al., 2021). Previous studies on *L. stoechas* have highlighted its anti-inflammatory effects. *L. stoechas* essential oil effectively reduced nitrite production in cell cultures (Zuzarte et al., 2013). In rat colitis and carrageenan-induced paw edema models, *L. stoechas* extract exhibited an anti-inflammatory impact, comparable to dexamethasone (Algieri et al., 2016). Similarly, we observed a noteworthy decrease in inflammatory cells eosinophils, neutrophils, and macrophages in 200 and 300 mg/kg *L. stoechas* groups. This reduction was comparable to that seen in the asthma group treated with dexamethasone, in contrast to the untreated asthma animals. Another study demonstrated that the hydroethanolic extract of the aerial part (branches, flowers, and leaves) of *L. stoechas* led to a noteworthy reduction in rats' paw edema volume (Yassine et al., 2016). In the current investigation, treatment involving *L. stoechas* demonstrated a notable reduction in allergen-induced cytokine expression, specifically *TNF-α* and *IL-6*, within the pulmonary region. Type 2 inflammation is recognized for its pivotal role in the pathogenesis of allergic asthma. Each of the type 2 cytokines performs multifaceted functions within the cascade of airway inflammation. Moreover, the secretion of mucins from airway epithelial cells can be induced by various inflammatory mediators, including TNF-α. This particular cytokine has been evidenced to elicit mucin release through the activation of nitric oxide synthase, as well as via NF-κB activation in human lung epithelial cells (Gurusamy et al., 2023). IL-6, a cytokine generated by inflammatory cells, is also synthesized by primary lung epithelial cells in response to diverse stimuli such as allergens, respiratory viruses, and physical exertion. While IL-6 is predominantly produced by cells of the innate immune system (e.g. macrophages, dendritic cells, mast cells, neutrophils), it is also expressed by B cells and, to a lesser extent, certain CD4 effector Th cells. The presence of IL-6 in the airways of individuals with asthma may not solely stem from ongoing inflammation but rather from the "activated" state of pulmonary epithelial cells (Rincon and Irvin, 2012). We have previously shown that pulmonary arterial hypertension and chronic airway diseases such as asthma and chronic obstructive pulmonary disease (COPD) share key pathological features, including inflammation, smooth muscle proliferation, and lung tissue remodeling (Gurusamy et al., 2023). Our current study's preliminary data also suggests the reversibility and improvement of lung structural changes in the 300 mg/kg *L. stoechas* treatment group compared to untreated asthma mice. This improvement in lung structural changes was comparable to that observed in dexamethasone-treated animals.

Glucocorticoids exert their anti-inflammatory effects through a combination of transrepression and transactivation mechanisms. This process involves the physical interaction between monomeric complexes of glucocorticoid and glucocorticoid receptor alpha (G–GRα) with NF-κB, as well as conformational changes resulting from the dimerization of the G–GRα complex (Gurusamy et al., 2016). In this study, we administered concentrations of 200 and 300 mg/kg of *L. stoechas* to treat allergic asthma in a BALB-C mouse model. Interestingly, the concentration of 300 mg/kg exhibited more pronounced reductions in the disease symptoms. While there may be variances in the mechanisms of action between glucocorticoids and *L. stoechas*, the current study showcases that a solution of *L. stoechas* at a dosage of 300 mg/kg, demonstrates nearly equivalent efficacy in mitigating OVA-induced airway inflammation, when juxtaposed with the hormonal anti-inflammatory agent dexamethasone. However, our study was subject to certain limitations. Foremost among these was our incomplete understanding of the precise molecular signaling pathways associated with *L. stoechas* extract and its efficacy in OVA-induced allergic asthma. Consequently, we did not comprehensively evaluate the wide spectrum of molecular mechanisms involved but rather focused on the primary objectives of the study. Further investigations are warranted to elucidate the therapeutic potential of this substance in the context of OVA-induced asthma models.

Our research outcomes decisively endorse the notion that *L. stoechas* harbors substantial promise as a therapeutic intervention to effectively impede the advancement of airway inflammation and remodeling. In light of these findings, our study underscores the potential of *L. stoechas* as a valuable herbal candidate for addressing bronchial inflammation, thereby mitigating the reduction in inflammatory cell counts in the context of acute lung inflammation and asthma. Nevertheless, further investigations are imperative to meticulously elucidate the precise mechanism through which *L. stoechas* operates in alleviating airway inflammation.

**Table 1:** Primer sequences used in this study

Primer	Sequence
*IL-6*	F: 5’-TGTGCAATGGCAATTCTGAT- 3’
	R: 5’- GGAAATTGGGGTAGGAGGGA- 3’
*TNF-α*	F: 5′- CCCCAAAGGGATGAGAAGTT- 3’
	R: 5′- GTGGGTGAGGAGCACGTAGT- 3’
*β-actin*	F: 5′- GGGAATGGGTCAGAAGGA -3’
	R: 5’-TTTGATGTCACGCACGATTT-3’
